# Association of number of siblings, birth order, and thinness in 3- to 12-year-old children: a population-based cross-sectional study in Shanghai, China

**DOI:** 10.1186/s12887-020-02261-z

**Published:** 2020-08-05

**Authors:** Tingting Yu, Chang Chen, Zhijuan Jin, You Yang, Yanrui Jiang, Li Hong, Xiaodan Yu, Hao Mei, Fan Jiang, Hong Huang, Shijian Liu, Xingming Jin

**Affiliations:** 1 School of Public Health, Shanghai Jiao Tong University School of Medicine, Shanghai, China; 2 Pediatric Translational Medicine Institute, Shanghai Children’s Medical Center, Shanghai Jiao Tong University School of Medicine, 1678 Dongfang Road, Shanghai, 200127 China; 3 Shanghai Key Laboratory of Children’s Environmental Health, Xinhua Hospital, Shanghai Jiao Tong University School of Medicine, 1665 Kongjiang Road, Shanghai, 200092 China; 4 Department of Developmental and Behavioral Pediatrics, Shanghai Children’s Medical Center, Shanghai Jiao Tong University School of Medicine, Shanghai, China; 5 Department of Clinical Nutrition, Shanghai Children’s Medical Center, Shanghai Jiao Tong University School of Medicine, Shanghai, China; 6 Department of Data Science, School of Population Health, University of Mississippi Medical Center, Jackson, Mississippi USA; 7 Department of Developmental and Behavioral Pediatrics, Shanghai Pubin Children Hospital, Shanghai, China

**Keywords:** Number of siblings, Birth order, Thinness, Children

## Abstract

**Background:**

Sibship size and structure have a significant association with overweight and obesity in children, but the relationship with thinness has not been fully studied and understood, especially in Asia. This study evaluated the associations among number of siblings, birth order, and childhood thinness and investigated the association of number of younger or older siblings with childhood thinness.

**Methods:**

In this study, we performed a population-based cross-sectional study among 84,075 3- to 12-year-old children in Shanghai using multistage stratified cluster random sampling. We defined grades 1, 2, and 3 thinness according to the body mass index cutoff points set by the International Obesity Task Force and used multinomial logistic regression models to estimate the odds ratio (OR) and 95% confidence interval (95% CI).

**Results:**

Compared with only children, for boys, children with two or more siblings were more likely to suffer from grade 2 (OR = 1.29, 95% CI 1.02, 1.64) and grade 3 thinness (OR = 1.60, 95% CI 1.07, 2.40); and the youngest child faced a higher risk of grade 2 (OR = 1.44, 95% CI 1.09, 1.90) and grade 3 thinness (OR = 1.53, 95% CI 1.01, 2.33). For girls, children with one sibling were more likely to suffer from grade 1 thinness (OR = 1.22, 95% CI 1.05, 1.42); the oldest child, middle child, and youngest child faced a higher risk of grade 1 (OR = 1.42, 95% CI 1.09, 1.84), grade 2 (OR = 1.26, 95% CI 1.03, 1.54), and grade 1 thinness (OR = 1.87, 95% CI 1.21, 2.88) respectively. There was no statistically significant relationship, however, between a larger number of younger or older siblings and childhood thinness.

**Conclusions:**

Regardless of sex, having either siblings or a higher birth order was positively associated with childhood thinness. The present study has suggested that future interventions to prevent childhood thinness should consider family background as an important factor, especially in multi-child-families.

## Background

Preschool- and school-age years are critical periods for a child’s growth and development. During these stages, children experience rapid but incomplete physical and psychological development, causing them to be the most vulnerable group in the population. Thus, children require more attention and support from family and society [1, 2]. With the rapid development of the economy and the advancement of urbanization, the double burden of obesity and thinness has become increasingly prominent in many developing countries [3–5]. Although the prevalence of overweight and obesity has received considerable attention in China, many researchers have shown that childhood thinness is also an important public health problem that cannot be ignored. In 2010, China had a thinness rate of 9.0% among children ages 6 to 17 years old, including 10.4% for boys and 7.3% for girls [6]. In Shanghai, the prevalence of thinness was 13.92% for boys and 18.45% for girls ages 3 to 12 years old [7].

Thinness is an indicator of recent undernutrition and eating disorders and often is associated with physical, mental, and intellectual development problems, as well as a higher risk of metabolic disease in adulthood [8–10]. Families play a vital role in the intervention of children with undernutrition, and studies have reported that family structure affects childhood physical development [11–13]. In one-child families, parents and grandparents pay all their attention to their single child or grandchild. Excessive doting by family members has resulted in childhood overweight and obesity [14]. In multi-child-families, however, with an increase in the number of siblings, parents’ time, energy, and financial resources are diluted among their children [15]. Studies have found that sibship composition is significantly associated with childhood obesity or undernutrition (thinness, stunting, or underweight) [13, 16–22]. Most studies have supported the finding that children who have siblings and older children have a lower risk of being overweight or obese [13, 16–19]. Results illustrating the associations of the number of siblings and birth order with undernutrition were inconsistent, however. Some studies found that a child’s risk of undernutrition was higher as the birth order or number of siblings increased [20–22]. A previous study reported that a larger number of siblings increased the odds ratio for thinness for girls but not for boys [23]. By contrast, one study showed no relationship between the number of siblings or birth order and thinness [24].

To change the demographic structure, China has introduced several family-planning policies, including the one-child policy, which was introduced nationwide in 1980 (except for ethnic minorities and rural families where the first child was a girl) but that was influenced by region, parental educational level, family economic level, and other factors during its implementation period; and the two-child policy, which was adopted for families with one parent as the only child in 2013 and then implemented nationwide in 2016 [25]. China became the country with the largest population of only children in the world (about 100 million) as a result of this one-child policy [26]. However, with the implementation of China’s two-child policy, family structure and personal relationships, especially sibling structure and relationships, became increasingly complicated and have had an unpredictable effect on childhood health. Therefore, after excluding the influence of early life nutrition, childhood living habits, and family economic level, which all were related to sibship composition and childhood thinness, this study evaluated the influence of sibship size and structure on childhood thinness and discerned whether sex interaction existed between them.

## Methods

### Study design and participants

**Fig. 1:**
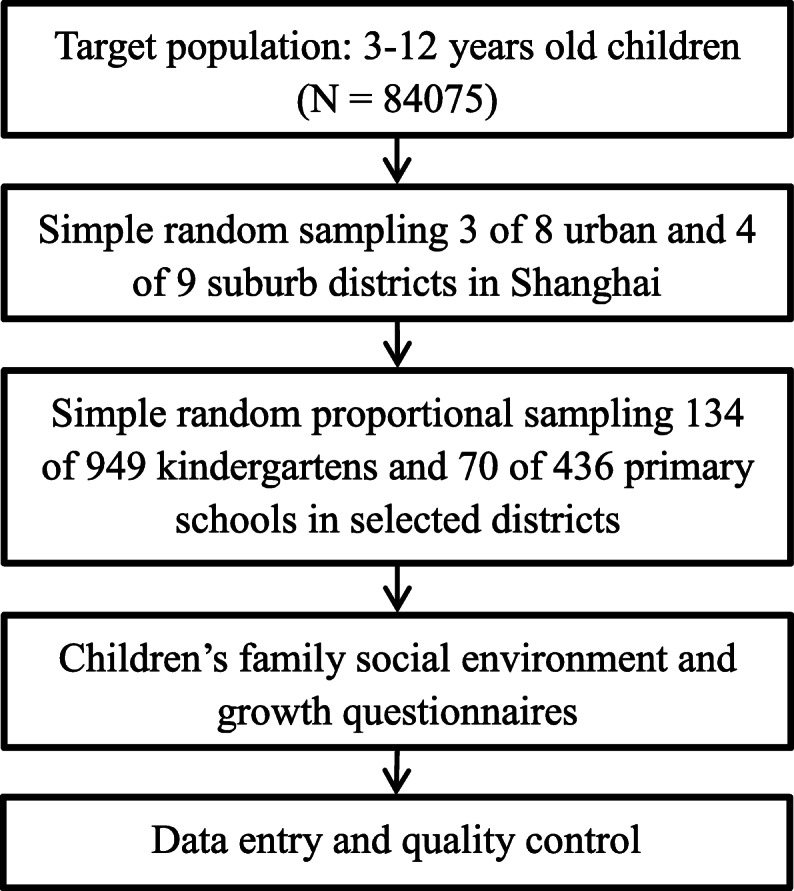
Study flowchart using multistage and stratified cluster random sampling

This investigation was based on a large school-based cross-sectional study that was part of a population survey of autism spectrum disorders led by the government. Relevant sampling methods have been described in a previous study [7] and are briefly stated as follows. This study was conducted using multistage, stratified cluster random sampling among children ages 3 to 12 years old in Shanghai, China, in June 2014. We randomly selected three urban districts and four suburban districts from a total of 17 districts across Shanghai. In total, 134 of 949 (14.12%) kindergarten schools, as well as 70 of 436 (16.06%) primary schools, were randomly sampled from a set of schools located in the selected districts (Fig. 1). In total, 84,075 of 576,621 (14.58%) children were recruited from these selected schools according to the proportion of students in each district to all of the sampled districts.

The child’s family, social environment, and growth questionnaires were administered to teachers, who accepted uniform training on completing, distributing, and collecting the questionnaires. Teachers informed their students to take the questionnaire home, and then the students’ parents were asked to complete the questionnaire to collect multilevel information on the child’s characteristics (e.g., age, sex, weight, height, number of siblings, birth order, birthweight, feeding pattern, parental ages at childbirth, workday TV time, Internet use time, and snacking frequency) and family structure (e.g., parental weight status, parental education level, family income and residential site). Then, the teachers collected the completed questionnaires and returned them to the investigators. Questionnaires with key information missing, including height, weight, number of siblings, or birth order, were excluded in the final analysis.

### Measurement

Body mass index (BMI, kg/m^2^) was calculated as weight (kg) divided by height (m) squared. Thinness, overweight, and obesity were defined according to the International Obesity Task Force–recommended age- and sex-specific cutoff points for children ages 2 to 18 years old. The BMI cutoffs for grades 1, 2, and 3 were < 18.5, < 17.0, and < 16.0 kg/m^2^, respectively, and the cutoff for overweight was ≥25.0 kg/m^2^. The cutoff for obesity was ≥30.0 kg/m^2^, and the cutoff for severe obesity was ≥35.0 kg/m^2^ [27, 28]. For adults, the weight status was categorized by BMI into underweight (< 18.5 kg/m^2^), normal weight (18.5–25.0 kg/m^2^), and overweight (≥25.0 kg/m^2^) classes, which included obesity and severe obesity as defined based on the World Health Organization cutoffs.

According to a previous study [17], we divided the number of siblings into three groups as follows: none (only child), one, and two or more siblings. We categorized birth order into four groups as follows: only child, oldest child, youngest child, and middle child. We included the number of younger or older siblings in three groups: none (only child), one, and two or more siblings. For birth order, the middle child represented children who had younger sibling(s) and older sibling(s). For the number of younger siblings, the one-sibling group or the two-or-more-siblings group represented the children who were the oldest child and who had either one or two or more younger siblings. For the number of older siblings, one or two or more siblings represented children who were the youngest child and who had either one or two or more older siblings.

Childhood characteristics included age (in years), sex (boy, girl), birthweight (< 2500, 2500–4000, or ≥ 4000 g), feeding pattern (breast-feeding, formulary-feeding, mixed-feeding), parental ages at childbirth (< 25, 25–34, or ≥ 35 years old), workday TV time (< 1, 1–3, or > 3 h/day), Internet use time (< 2, 2–4, or > 4 h/day), and snacking frequency (0, 1–3, or > 3 times/day) were considered as potential prenatal confounding factors [22, 24, 29]. Family characteristics included parental education level, which was divided into low (illiterate, primary school, or junior high school), middle (senior high school, technical school, or college), and high (undergraduate or above). Family income was categorized into three groups as follows: low (< 10,000, 10,000–30,000, or 30,000–50,000 Chinese yuan), middle (50,000–100,000, 100,000–150,000, or 150,000–200,000 Chinese yuan), and high (200,000–300,000, 300,000–1,000,000, and > 1,000,000 Chinese yuan), according to a social science definition [30]. Residential site was defined as urban or suburban residents according to the participants’ living district.

### Statistical analysis

We used EpiData 3.1 (EpiData Association, Odense, Denmark) for data entry and applied a logic error check. To ensure the reliability, consistency, and correctness of inputted data, we randomly sampled 15% of questionnaires for repeat data entry. We obtained verbal consent from all participants and their parents before investigation. This study was approved by the Institutional Review Boards of the Shanghai Municipal Commission of Health and Family Planning.

All statistical analyses were conducted using the software package IBM SPSS Statistics (version 24.0). We computed sampling weights using inverse probability weighting, which represented the inverse of the combined selection probability in each sampling stage. Sample weight (*Wt_Sample*) was the product of the sampling weights and the nonresponse weight, which was calculated by the following equation:

1 $$ Wt\_ Sample= Wt\_ Strat 1\times Wt\_ Strat 2\times Wt\_ NR, $$

where *Wt_Strat1* is the inverse probability of an “urban district” or “suburban district” being selected in the central urban or suburban districts stratum in Shanghai, *Wt_Strat2* represents the inverse probability of a “kindergarten” or “primary school” being selected in the kindergarten or primary school stratum in each selected district, and *Wt_NR* is the inverse probability of the nonresponse rate for questionnaires in each of the selected districts.

We used the Chi-square tests to compare the distribution of childhood and family characteristics, as well as prevalence of thinness among the groups for different numbers of siblings, birth order, number of younger siblings, and number of older siblings. Combing with LOGISTIC module in complex sampling, which considered sample weight and sampling method, we used multinomial logistic regression models to calculate the OR and 95% CI of the number of siblings, birth order, number of younger siblings, and number of older siblings for grades 1, 2, and 3 thinness among boys and girls. We made additional adjustments for the multinomial regression models, including model I: adjusted for age, which was related to the BMI category; model II: adjusted for age and childhood characteristics, including birthweight, feeding pattern, parental age at childbirth, workday TV time, Internet use time, and snacking frequency, which were reported to be associated with sibship composition and BMI category; and model III: adjusted for age, childhood characteristics, and family characteristics, including parental weight status, parental education level, family income, and residential site, which could reflect the family resources for children to some degree. The statistical significance was defined as a *P*-value < 0.05 by a two-tailed test.

## Results

A total of 84,075 questionnaires were distributed to participants ages 3 to 12 years old, and 81,384 completed questionnaires were collected with a response rate of 96.80%. In total, 13,810 children (16.97%) were excluded, among which 8949 (11.00%) had incomplete height or weight data, and 4861 (5.97%) had no data on number of siblings and birth order. We included 67,574 children in our final analysis, including 35,835 boys (53.03%) and 31,739 girls (46.97%).

Table 1 shows the characteristics of study participants arranged by the number of siblings. Overall, the number of children with no siblings (only child), one sibling, and two or more siblings were 49,097 (72.66%), 6852 (10.14%), and 11,625 (17.20%), respectively. The average age (mean ± SD, standard deviations) of only children, those with one sibling, and those with two or more siblings was 7.03 ± 2.30, 6.99 ± 2.25, and 7.41 ± 2.19 years, respectively (not shown in the table). The proportion of boys in each number of sibling groups was higher than that of girls, especially in the one-child category (*p* < 0.001). Low birthweight (*p* = 0.029) and breast-feeding (*p* < 0.001) and mothers (*p* < 0.001) or fathers (*p* < 0.001) aged at childbirth younger than 25 years old were more common in the two-or-more-siblings group. Workday TV time (*p* < 0.001), Internet use time (*p* < 0.001), and snacking frequency (*p* < 0.001) were statistically different in the groups with different numbers of siblings.

**Table 1:** Characteristics of study participants by the number of siblings

Variables	None (only child)		One		Two or more		Total		χ^2^	*P* value^a^
	N	%	N	%	N	%	N	%		
Sex									32.94	< 0.001
Boy	26,351	53.67	3457	50.45	6027	51.85	35,835	53.03		
Girl	22,746	46.33	3395	49.55	5598	48.15	31,739	46.97		
Total	49,097	100.00	6852	100.00	11,625	100.00	67,574	100.00		
Birth weight (g)									4.77	0.029
< 2500	1631	3.32	417	6.09	939	8.08	2987	4.42		
2500–4000	41,584	84.70	5532	80.74	8712	74.94	55,828	82.62		
≥ 4000	4883	9.95	722	10.54	1393	11.98	6998	10.36		
Missing	999	2.03	181	2.64	581	5.00	1761	2.61		
Total	49,097	100.00	6852	100.00	11,625	100.00	67,574	100.00		
Feeding patterns									8475.14	< 0.001
Breast Feeding	21,893	44.59	3546	51.75	7411	63.75	32,850	48.61		
Formulary Feeding	7957	16.21	1054	15.38	1674	14.40	10,685	15.81		
Mixed Feeding	18,965	38.63	2194	32.02	2391	20.57	23,550	34.85		
Missing	282	0.57	58	0.85	149	1.28	489	0.72		
Total	49,097	100.00	6852	100.00	11,625	100.00	67,574	100.00		
Workday TV time (hour/day)									41,614.76	< 0.001
< 1	29,037	59.14	3762	54.90	5484	47.17	38,283	56.65		
1–3	18,353	37.38	2843	41.49	5534	47.60	26,730	39.56		
> 3	990	2.02	135	1.97	303	2.61	1428	2.11		
Missing	717	1.46	112	1.63	304	2.62	1133	1.68		
Total	49,097	100.00	6852	100.00	11,625	100.00	67,574	100.00		
Internet use time (hour/week)									33,821.76	< 0.001
< 2	30,086	61.28	4518	65.94	7930	68.22	42,534	62.94		
2–4	11,235	22.88	1346	19.64	1742	14.98	14,323	21.20		
≥ 4	6301	12.83	717	10.46	769	6.62	7787	11.52		
Missing	1475	3.00	271	3.96	1184	10.18	2930	4.34		
Total	49,097	100.00	6852	100.00	11,625	100.00	67,574	100.00		
Snack frequency (times/day)									37,680.69	< 0.001
0	14,854	30.25	1951	28.47	2716	23.36	19,521	28.89		
1–3	30,643	62.41	4355	63.56	7724	66.44	42,722	63.22		
> 3	2994	6.10	458	6.68	846	7.28	4298	6.36		
Missing	606	1.23	88	1.28	339	2.92	1033	1.53		
Total	49,097	100.00	6852	100.00	11,625	100.00	67,574	100.00		
Father’s age at child birth (years)									976.81	< 0.001
< 25	7217	14.70	1119	16.33	2263	19.47	10,599	15.69		
25–34	33,800	68.84	4078	59.52	6264	53.88	44,142	65.32		
≥ 35	4918	10.02	1142	16.67	1902	16.36	7962	11.78		
Missing	3162	6.44	513	7.49	1196	10.29	4871	7.21		
Total	49,097	100.00	6852	100.00	11,625	100.00	67,574	100.00		
Mother’s age at child birth (years)									1518.61	< 0.001
< 25	15,672	31.92	2092	30.53	3872	33.31	21,636	32.02		
25–34	30,882	62.90	3985	58.16	5933	51.04	40,800	60.38		
≥ 35	1409	2.87	571	8.33	1173	10.09	3153	4.67		
Missing	1134	2.31	204	2.98	647	5.57	1985	2.94		
Total	49,097	100.00	6852	100.00	11,625	100.00	67,574	100.00		

^a^*P* Value from Chi-squared test

Family characteristics according to the number of siblings are shown in Table 2. In the only-child group, more children had fathers (*p* < 0.001) and mothers (*p* < 0.001) who were underweight and more children were from urban residential families (*p* < 0.001). Most of the only children had a highly educated father (*p* < 0.001) or mother (*p* < 0.001) and had higher family income (*p* < 0.001) than that of the children with siblings.

**Table 2:** Family characteristics of study participants by the number of siblings

Variables	None (only child)		One		Two or more		Total		χ^2^	*P v*alue^a^
	N	%	N	%	N	%	N	%		
Father’s weight status									19.60	0.001
Underweight	1594	3.25	203	2.96	288	2.48	2085	3.09		
Normal	30,935	63.01	4353	63.53	7394	63.60	42,682	63.16		
Overweight	12,667	25.80	1788	26.09	2946	25.34	17,401	25.75		
Missing	3901	7.95	508	7.41	997	8.58	5406	8.00		
Total	49,097	100.00	6852	100.00	11,625	100.00	67,574	100.00		
Father’s education level									10,349.70	< 0.001
Low	8524	17.36	2416	35.26	7275	62.58	18,215	26.96		
Middle	22,081	44.97	2386	34.82	3137	26.98	27,604	40.85		
High	18,292	37.26	2023	29.52	1162	10.00	21,477	31.78		
Missing	200	0.41	27	0.39	51	0.44	278	0.41		
Total	49,097	100.00	6852	100.00	11,625	100.00	67,574	100.00		
Mother’s weight status									509.04	< 0.001
Underweight	5260	10.71	579	8.45	726	6.25	6565	9.72		
Normal	35,611	72.53	4982	72.71	8193	70.48	48,786	72.20		
Overweight	4794	9.76	840	12.26	1807	15.54	7441	11.01		
Missing	3432	6.99	451	6.58	899	7.73	4782	7.08		
Total	49,097	100.00	6852	100.00	11,625	100.00	67,574	100.00		
Mother’s education level									12,096.00	< 0.001
Low	9842	20.05	2691	39.27	8306	71.45	20,839	30.84		
Middle	22,742	46.32	2511	36.65	2475	21.29	27,728	41.03		
High	16,450	33.51	1632	23.82	823	7.08	18,905	27.98		
Missing	63	0.13	18	0.26	21	0.18	102	0.15		
Total	49,097	100.00	6852	100.00	11,625	100.00	67,574	100.00		
Family income									3362.13	< 0.001
Low	10,257	20.89	1828	26.68	5368	46.18	17,453	25.83		
Middle	29,251	59.58	3322	48.48	4862	41.82	37,435	55.40		
High	9563	19.48	1694	24.72	1388	11.94	12,645	18.71		
Missing	26	0.05	8	0.12	7	0.06	41	0.06		
Total	49,097	100.00	6852	100.00	11,625	100.00	67,574	100.00		
Residential site									1241.61	< 0.001
Suburban	36,765	74.88	5567	81.25	10,421	89.64	52,753	78.07		
Urban	12,313	25.08	1284	18.74	1200	10.32	14,797	21.90		
Missing	19	0.04	1	0.01	4	0.03	24	0.04		
Total	49,097	100.00	6852	100.00	11,625	100.00	67,574	100.00		

^a^*P* Value from Chi-squared test

We calculated the prevalence of thinness in relation to the number of siblings, birth order, and number of younger or older siblings (the distribution is shown in Table 3). In general, the prevalence of thinness of only children (14.96%) was lower than that of children with siblings (one sibling: 18.18%; two or more siblings: 17.45%). In the only-, oldest-, middle-, and youngest-child groups, the prevalence of thinness increased as birth order increased (14.96, 17.73, 17.11, and 19.51%, respectively). In the groups with different numbers of younger or older siblings, thinness was more common in the oldest child with two or more younger siblings (18.31%) or in the youngest child with one older sibling (17.53%).

**Table 3:** Distribution of prevalence of thinness by sibship size or structure

Variables	Total sample	Grade 1 thinness		Grade 2 thinness		Grade 3 thinness		Total thinness	
	N	N	%	N	%	N	%	N	%
Number of siblings									
None (only child)	49,097	4884	9.95	1469	2.99	993	2.02	7346	14.96
One	6852	784	11.44	258	3.77	204	2.98	1246	18.18
Two or more	11,625	1128	9.70	423	3.64	478	4.11	2029	17.45
Total	67,574	6796	10.06	2150	3.18	1675	2.48	10,621	15.72
Birth order									
Only child	49,097	4884	9.95	1469	2.99	993	2.02	7346	14.96
Oldest child	7941	855	10.77	285	3.59	268	3.37	1408	17.73
Middle	6696	638	9.53	240	3.58	268	4.00	1146	17.11
Youngest child	3162	359	11.35	142	4.49	116	3.67	617	19.51
Missing	678	60	8.85	14	2.06	30	4.42	104	15.34
Total	67,574	6796	10.06	2150	3.18	1675	2.48	10,621	15.72
Number of younger siblings									
None (only child)	49,097	4884	9.95	1469	2.99	993	2.02	7346	14.96
One (oldest child)	3691	421	11.41	119	3.22	90	2.44	630	17.07
Two or more (oldest child)	4250	434	10.21	166	3.91	178	4.19	778	18.31
Total	57,038	5739	10.06	1754	3.08	1261	2.21	8754	15.35
Number of older siblings									
None (only child)	49,097	4884	9.95	1469	2.99	993	2.02	7346	14.96
One (youngest child)	2968	342	11.52	134	4.51	107	3.61	583	19.64
Two or more (youngest child)	194	17	8.76	8	4.12	9	4.64	34	17.53
Total	52,259	5243	10.03	1611	3.08	1109	2.12	7963	15.24

**Fig. 2:**
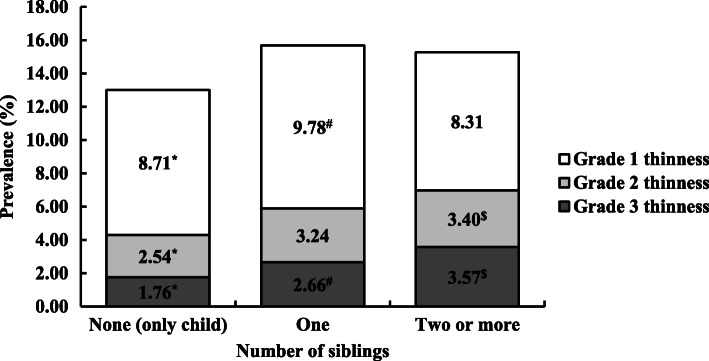
Distribution of the prevalence of grades 1, 2, and 3 thinness in boys. ^*^Statistically significant difference in prevalence between 0 (only child) and 1 sibling (χ2 test): *p* < 0.05; ^#^Statistically significant difference in prevalence between 1 and ≥ 2 siblings (χ2 test): *p* < 0.05; ^$^Statistically significant difference in prevalence between 0 (only child) and ≥ 2 siblings (χ2 test): *p* < 0.05.

**Fig. 3:**
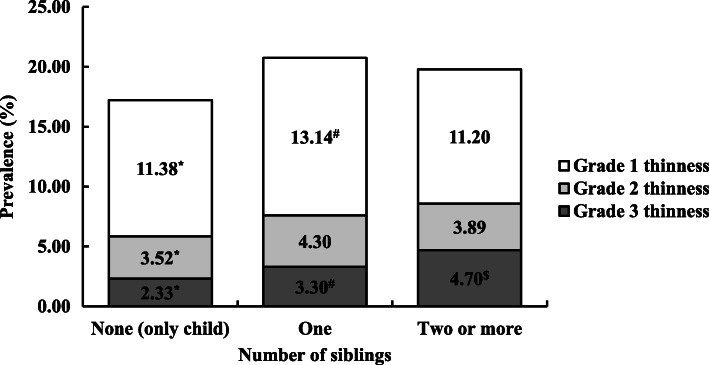
Distribution of the prevalence of grades 1, 2, and 3 thinness in girls. ^*^Statistically significant difference in prevalence between 0 (only child) and 1 sibling (χ2 test): *p* < 0.05; ^#^Statistically significant difference in prevalence between 1 and ≥ 2 siblings (χ2 test): *p* < 0.05; ^$^Statistically significant difference in prevalence between 0 (only child) and ≥ 2 siblings (χ2 test): *p* < 0.05

In the only-child, one-sibling, and two-or-more-siblings groups of boys (Fig. 2), the prevalence of grade 2 thinness (2.54, 3.24, 3.40%, respectively) and grade 3 thinness (1.76, 2.66, 3.57%, respectively) was highest in the two-or-more-siblings group, and the prevalence of grade 1 thinness (8.71, 9.78, 8.31%, respectively) was highest in the one-sibling group. As for girls (Fig. 3), the prevalence of grade 1 thinness (11.38, 13.14, 11.20%, respectively) and grade 3 thinness (2.33, 3.30, 4.70%, respectively) was highest in the one-sibling and two-or-more-siblings group, respectively, but not in the only-child group. This was similar with the prevalence for boys, except that the prevalence of grade 2 thinness (3.52, 4.30, 3.89%, respectively) was highest for boys in the one-sibling group. Overall, girls were more likely to be thin than boys.

Crude and adjusted ORs of the number of siblings, birth order, and number of younger or older siblings for thinness among boys and girls are presented in Tables 4 and 5, respectively. Among boys, in model III, children with two or more siblings were more likely to suffer from grade 2 (OR = 1.29, 95% CI 1.02, 1.64) and grade 3 thinness (OR = 1.60, 95% CI 1.07, 2.40) compared with only child; and youngest children faced a higher risk of grade 2 (OR = 1.44, 95% CI 1.09, 1.90) and grade 3 thinness (OR = 1.53, 95% CI 1.01, 2.33). Although there was no significant relationship with thinness for a larger number of younger or older siblings, in two-child families, the younger child had a higher OR for grade 3 thinness (OR = 1.57, 95% CI 1.18, 2.09), and in three-or-more-children families, the oldest child faced a higher risk of grade 2 thinness (OR = 2.00, 95% CI 1.47, 1.72). Among girls, in model III, children with one sibling were more likely to suffer from grade 1 thinness (OR = 1.22, 95% CI 1.05, 1.42); and the oldest child, middle child, and youngest child faced a higher risk of grade 1 (OR = 1.42, 95% CI 1.09, 1.84), grade 2 (OR = 1.26, 95% CI 1.03, 1.54), and grade 1 thinness (OR = 1.87, 95% CI 1.21, 2.88), respectively. In families with children who had siblings, the youngest child with one older sibling had a higher risk of grade 1 (OR = 1.38, 95% CI 1.05, 1.81) and grade 3 thinness (OR = 1.84, 95% CI 1.15, 2.93).

**Table 4:** Multinomial logistical regression of sibship size or structure for thinness among boys

	Total N	Grade 1 thinness			Grade 2 thinness			Grade 3 thinness		
		Model I^a^	Model II^b^	Model III^c^	Model I^a^	Model II^b^	Model III^c^	Model I^a^	Model II^b^	Model III^c^
Number of siblings										
None (only child)	26,351	1.00 (Ref)	1.00 (Ref)	1.00 (Ref)	1.00 (Ref)	1.00 (Ref)	1.00 (Ref)	1.00 (Ref)	1.00 (Ref)	1.00 (Ref)
One	3457	1.13 (1.03, 1.24)*	1.11 (1.00, 1.24)	1.10 (0.98, 1.24)	1.27 (1.02, 1.60)*	1.34 (1.07, 1.69)*	1.28 (1.00, 1.63)	1.48 (1.08, 2.03)*	1.38 (0.96, 1.99)	1.31 (0.89, 1.92)
Two or more	6027	1.00 (0.88, 1.12)	0.96 (0.83, 1.12)	0.96 (0.80, 1.16)	1.41 (1.19, 1.67)*	1.46 (1.17, 1.82)*	1.29 (1.02, 1.64)*	2.16 (1.61, 2.90)*	1.88 (1.30, 2.72)*	1.60 (1.07, 2.40)*
Birth order										
Only child	26,351	1.00 (Ref)	1.00 (Ref)	1.00 (Ref)	1.00 (Ref)	1.00 (Ref)	1.00 (Ref)	1.00 (Ref)	1.00 (Ref)	1.00 (Ref)
Oldest child	3331	0.95 (0.81, 1.12)	0.91 (0.75, 1.10)	0.89 (0.73, 1.08)	1.33 (1.07, 1.64)*	1.28 (1.02, 1.61)*	1.32 (1.03, 1.70)*	1.57 (1.20, 2.04)*	1.34 (0.99, 1.82)	1.26 (0.90, 1.77)
Middle child	3923	1.09 (0.92, 1.30)	1.08 (0.86, 1.35)	1.11 (0.86, 1.44)	1.36 (1.10, 1.68)*	1.45 (1.08, 1.93)*	1.20 (0.93, 1.56)	2.14 (1.57, 2.94)*	1.97 (1.29, 3.02)*	1.61 (0.98, 2.64)
Youngest child	1869	1.13 (0.96, 1.32)	1.16 (0.94, 1.44)	1.17 (0.95, 1.45)	1.54 (1.14, 2.08)*	1.73 (1.30, 2.28)*	1.44 (1.09, 1.90)*	1.82 (1.28, 2.59)*	1.74 (1.20, 2.52)*	1.53 (1.01, 2.33)*
Number of younger siblings										
None (only child)	26,351	1.00 (Ref)	1.00 (Ref)	1.00 (Ref)	1.00 (Ref)	1.00 (Ref)	1.00 (Ref)	1.00 (Ref)	1.00 (Ref)	1.00 (Ref)
One (oldest child)	1614	1.09 (0.86, 1.37)	1.04 (0.79, 1.38)	1.03 (0.77, 1.39)	0.94 (0.69, 1.28)	0.91 (0.64, 1.28)	1.03 (0.73, 1.45)	1.13 (0.71, 1.81)	1.04 (0.64, 1.68)	1.03 (0.63, 1.68)
Two or more (oldest child)	1717	0.81 (0.61, 1.08)	0.78 (0.60, 1.02)	0.74 (0.55, 0.99)	1.70 (1.30, 2.23)*	1.64 (1.20, 2.23)*	1.59 (1.12, 2.28)*	2.00 (1.47, 2.72)*	1.60 (1.06, 2.41)*	1.44 (0.90, 2.29)
Number of older siblings										
None (only child)	26,351	1.00 (Ref)	1.00 (Ref)	1.00 (Ref)	1.00 (Ref)	1.00 (Ref)	1.00 (Ref)	1.00 (Ref)	1.00 (Ref)	1.00 (Ref)
One (youngest child)	1738	1.17 (0.96,1.42)	1.20 (0.93, 1.56)	1.19 (0.92, 1.55)	1.60 (1.18, 2.16)*	1.82 (1.35, 2.46)*	1.57 (1.18, 2.09)*	1.77 (1.23, 2.56)*	1.70 (1.10, 2.62)*	1.52 (0.91, 2.53)
Two or more (youngest child)	131	0.61 (0.14, 2.61)	0.66 (0.13, 3.24)	0.74 (0.14, 3.86)	0.81 (0.29, 2.26)	0.89 (0.20, 3.98)	0.93 (0.22, 4.01)	2.53 (0.82, 7.77)	2.91 (0.73, 11.63)	2.47 (0.66, 9.26)

Ref: reference category

^a^ Model I: adjusted for age

^b^ Model II: adjusted for age and childhood characteristics (birthweight, feeding patterns, parental age at child birth, workday TV time, internet use time, snacking frequency)

^c^ Model III: adjusted for age, childhood characteristics and family characteristics (parental weight status, parental educational level, family income and residential site)

*p < 0.05

**Table 5:** Multinomial logistical regression of sibship size or structure for thinness among girls

	Total N	Grade 1 thinness			Grade 2 thinness			Grade 3 thinness		
		Model I^a^	Model II^b^	Model III^c^	Model I^a^	Model II^b^	Model III^c^	Model I^a^	Model II^b^	Model III^c^
Number of siblings										
None (only child)	22746	1.00 (Ref)	1.00 (Ref)	1.00 (Ref)	1.00 (Ref)	1.00 (Ref)	1.00 (Ref)	1.00 (Ref)	1.00 (Ref)	1.00 (Ref)
One	3395	1.21 (1.06,1.36)*	1.20 (1.04, 1.39)*	1.22 (1.05, 1.42)*	1.26 (1.02, 1.55)*	1.23 (0.94, 1.60)	1.27 (0.94, 1.70)	1.56 (1.15, 2.11)*	1.06 (1.17, 2.17)*	1.39 (0.97, 1.99)
Two or more	5598	1.03 (0.91, 1.17)	0.96 (0.81, 1.14)	0.97 (0.78, 1.20)	1.18 (0.98, 1.43)	1.19 (1.01, 1.40)*	1.16 (0.97, 1.40)	2.25 (1.68, 3.02)*	2.02 (1.29, 3.15)*	1.49 (0.99, 2.24)
Birth order										
Only child	22746	1.00 (Ref)	1.00 (Ref)	1.00 (Ref)	1.00 (Ref)	1.00 (Ref)	1.00 (Ref)	1.00 (Ref)	1.00 (Ref)	1.00 (Ref)
Oldest child	4610	1.14 (1.04, 1.25)*	1.08 (0.96, 1.23)	1.08 (0.92, 1.27)	1.07 (0.87, 1.32)	1.12 (0.87, 1.45)	1.12 (0.84, 1.49)	1.81 (1.22, 2.67)*	1.58 (1.03, 2.44)*	1.30 (0.88, 1.93)
Middle child	2773	0.94 (0.74, 1.19)	0.87 (0.67, 1.13)	0.90 (0.69, 1.19)	1.28 (1.04, 1.58)*	1.28 (1.01, 1.62)*	1.26 (1.03, 1.54)*	2.21 (1.71, 2.86)*	2.07 (1.39, 3.07)*	1.49 (0.99, 2.25)
Youngest child	1293	1.33 (1.10, 1.62)*	1.37 (1.08, 1.73)*	1.42 (1.09, 1.84)*	1.70 (1.24, 2.34)*	1.52 (0.94, 2.46)	1.61 (0.97, 2.66)	2.08 (1.48, 2.92)*	2.38 (1.63, 3.48)*	1.87 (1.21, 2.88)*
Number of younger siblings										
None (only child)	22,746	1.00 (Ref)	1.00 (Ref)	1.00 (Ref)	1.00 (Ref)	1.00 (Ref)	1.00 (Ref)	1.00 (Ref)	1.00 (Ref)	1.00 (Ref)
One (oldest child)	2077	1.14 (0.98, 1.31)	1.11 (0.94, 1.31)	1.13 (0.94, 1.35)	1.07 (0.80, 1.43)	1.13 (0.82, 1.56)	1.15 (0.79, 1.66)	1.24 (0.83, 1.85)	1.18 (0.78, 1.78)	1.11 (0.67, 1.84)
Two or more (oldest child)	2533	1.14 (1.02, 1.29)*	1.06 (0.91, 1.24)	1.04 (0.85, 1.27)	1.06 (0.81, 1.38)	1.11 (0.83, 1.49)	1.09 (0.81, 1.46)	2.27 (1.46, 3.54)*	1.95 (1.13, 3.36)*	1.46 (0.88, 2.40)
Number of older siblings										
None (only child)	22,746	1.00 (Ref)	1.00 (Ref)	1.00 (Ref)	1.00 (Ref)	1.00 (Ref)	1.00 (Ref)	1.00 (Ref)	1.00 (Ref)	1.00 (Ref)
One (youngest child)	1230	1.32 (1.08, 1.61)*	1.36 (1.07, 1.72)*	1.38 (1.05, 1.81)*	1.65 (1.21, 2.25)*	1.45 (0.89, 2.39)	1.60 (0.95, 2.67)	2.07 (1.41, 3.02)*	2.41 (1.59, 3.63)*	1.84 (1.15, 2.93)*
Two or more (youngest child)	63	1.70 (0.94, 3.07)	1.80 (0.83, 3.90)	1.97 (0.91, 4.25)	2.96 (0.99, 8.85)	3.60 (1.20, 10.78)*	3.37 (1.12, 10.13)*	2.50 (0.76, 8.16)	2.21 (0.46, 10.55)	1.79 (0.38, 8.52)

Ref: reference category

^a^ Model I: adjusted for age

^b^ Model II: adjusted for age and childhood characteristics (birthweight, feeding patterns, parental age at child birth, workday TV time, internet use time, snacking frequency)

^c^ Model III: adjusted for age, childhood characteristics and family characteristics (parental weight status, parental educational level, family income and residential site)

*p < 0.05

Combining with result of the analysis of the association between sibship size or structure and thinness in the total samples (See Supplementary Table A1, Additional File 1) and in the samples of different genders (Tables 4 and 5) and results of sex-interaction in the multinomial logistic regression models (See Supplementary Table A2, Additional File 1), we found that those children with siblings or having a high birth order faced a higher risk of thinness, and youngest brothers and sisters were at greater risk of thinness in families with two children. Notably, this difference was in families with three or more children, and the oldest brothers and the youngest sisters tended to be more prone to thinness. These associations did not change in children with different gender, showing that there was not interaction effect between sibship size or structure and sex on thinness.

## Discussion

Our results showed that sibling children had a higher OR for thinness compared with only children. Few studies have reported that many siblings are a risk factor for thinness. However, one study found that having siblings increased ORs for childhood underweight, especially when a malnourished sibling lived within the household [29]. Other studies indicated that having a larger number of siblings was associated with a more significant decrease in BMI [18, 31]. One study, however, reported that there was no association between the number of siblings and thinness [24], whereas another study found that thinness was more common in girls than in boys [23]. One possible explanation is the effect of behavior and interaction among family members. On the one hand, upbringing and available resources for nutrition are different for children with different numbers of siblings, which may affect childhood weight status. A previous study reported that children with siblings faced a higher malnutrition risk [32]. Moreover, the nutritive value of diets for each child in small families was higher than that in large families, and children with siblings had significantly lower protein intake than only children [32]. It also has been reported that higher protein intake is associated with a lower risk of thinness [33]. On the other hand, additional sibling(s) enhanced interactions between children, and previous studies have identified a relationship between physical activity and siblings [34]. Moreover, children with siblings spent more time engaged in afterschool sports or household chores than only children [18]. In this study, when we adjusted sedentary behavior, including workday TV time and Internet use time, the positive association of the number of siblings with childhood thinness remained.

Regarding birth order, a higher birth order has been reported to significantly increase ORs for undernutrition [20–22, 35], which is consistent with our results. In contrast, some studies have not found a relationship between BMI or thinness and birth order [24, 31]. Thus, the association of birth order with childhood thinness remains unclear; however, differences in fetal nutrition and changes in some factors related to growth development in early life may explain this outcome. With increasing pregnancies and the expansion of household size, child- and family-related factors may have changed, such as birthweight and prenatal weight. The relationship between birth order and thinness, however, remained after adjusting for these factors. Thus, factors other than variates in the present study could be associated with the relationship between birth order and thinness. In addition, one study reported that firstborn children had lower birthweights than their younger siblings, yet they tended to be more sensitive to factors that could encourage growth [36].

In our study, there was no relationship between a greater number of younger or older siblings and thinness. In two-child families, however, younger children faced a higher risk of thinness, which may have been the result of being at a competitive disadvantage compared with their older siblings. Additionally, these children appeared to have spent more time engaging in physical activity as a result of imitating the behaviors of their older siblings [37].

Children with siblings or with a higher birth order were more likely to be thin in both boys and girls. The results showed, however, that as a family’s resource-relevant confounding factors adjusted the logistics model, the relationship between two or more siblings and thinness did not exist in girls but remained in boys. Therefore, girls may get less nutritional resources than boys in families with three or more children. Currently, there is no biological mechanism for the influence of family structure on children’s weight status. Most studies have discussed the family structure, which may lead to differences in children’s lifestyle, mainly including nutritional access and family interactions. Future studies related to family structure and physical development should pay attention to the availability of nutrition and physical and emotion interaction with parents and siblings among preschool and school-age children.

The strengths of our study are as follows. First, we evaluated the relationship between the number of siblings and birth order and childhood thinness. To our knowledge, no related study has been carried out in China. Second, the large sample size and multistage cluster random sampling employed ensured the use of representative data. Therefore, we had the ability to examine the influence of number of siblings and birth order on childhood thinness. Finally, we examined the relationship of the number of older or younger siblings with thinness, which previously had not been studied. This study also had some limitations. Shanghai has a high socioeconomic level; thus, samples from this study did not represent China as a whole. In addition, the implementation of the two-child policy may change the median birth interval of Chinese children, and the length of the birth interval has an impact on the status of a child’s weight [29]. We note that this information was not available for our study. Although we did include sedentary behaviors factors, including workday TV time and Internet use time, as well as dietary factors like snacking frequency as confounding factors into the adjusted logistic regression model, other potential confounding factors, including physical activity, nutrition intake, sleeping habits, emotional assessment, and parent–child relationship, that had an important effect on childhood status were not adjusted in this study. In fact, a recent study reported that the engagement level of family members was positively associated with a child’s diet quality [38]. Additionally, the collection of height and weight data of children according to self-reports may have affected the definition of weight status to some extent. Our findings may apply only to children between 3 and 12 years of age, and therefore, we cannot discern whether the number of siblings or birth order will have lasting effects on a child’s future health.

## Conclusions

In general, as the number of siblings and birth order increased, a child’s risk of thinness also increased. In two-child families, the youngest child was more likely to be thin. In addition to helping researchers better identify children at risk of thinness, our findings also provide a reference for the implementation and social policy formulation of child thinness intervention. On basis of these findings, when formulating policies for the allocation of social resources, developing countries should be more inclined toward families with two or more children to improve access to nutrition among these children, especially those in the lower level economic regions. At the same time, in terms of parenting styles, methods should be advocated that more reasonably plan nutrition intake and interaction among children with different birth orders.

## Supplementary information

**Additional file 1: Table A1.** Multinomial logistical regression of sibship size or structure for thinness. **Table A2.** Interaction effect between sibship size or structure and sex for thinness by using multinomial logistical regression.

## Data Availability

All data generated or analysed during this study are included in this published article and its supplementary information files.

## Abbreviations

BMI: Body mass index
SD: Standard deviations
OR: Odds ratio
CI: Confidence interval
