# Supplementary material for: Association of number of siblings, birth order, and thinness in 3- to 12-year-old children: a population-based cross-sectional study in Shanghai, China
Source: BMC Pediatr. 2020 Aug 5;20:367. doi: 10.1186/s12887-020-02261-z (PMC7405454; doi:10.1186/s12887-020-02261-z)
Supplement: Supplementary file 1 — Additional file 1: Table A1. Multinomial logistical regression of sibship size or structure for thinness. Table A2. Interaction effect between sibship size or structure and sex for thinness by using multinomial logistical regression. [file 12887_2020_2261_MOESM1_ESM.docx]

**Additional file 1:**

**Table A1** Multinomial logistical regression of sibship size or structure for thinness

|  | Total N | Grade 1 thinness | | |  | Grade 2 thinness | | |  | Grade 3 thinness | | |
| --- | --- | --- | --- | --- | --- | --- | --- | --- | --- | --- | --- | --- |
|  |  | Model I | Model II | Model III |  | Model I | Model II | Model III |  | Model I | Model II | Model III |
| Number of siblings |  |  |  |  |  |  |  |  |  |  |  |  |
| None (only child) | 49097 | 1.00 (Ref) | 1.00 (Ref) | 1.00 (Ref) |  | 1.00 (Ref) | 1.00 (Ref) | 1.00 (Ref) |  | 1.00 (Ref) | 1.00 (Ref) | 1.00 (Ref) |
| One | 6852 | 1.17 (1.08, 1.27)* | 1.16 (1.06, 1.27)* | 1.16 (1.06, 1.28)* |  | 1.27 (1.07, 1.50)* | 1.28 (1.06, 1.53)* | 1.27 (1.03, 1.57)* |  | 1.52 (1.29, 1.79)* | 1.50 (1.28, 1.74)* | 1.35 (1.15, 1.59)* |
| Two or more | 11625 | 1.02 (0.92, 1.12) | 0.96 (0.86, 1.07) | 0.97 (0.85, 1.10) |  | 1.28 (1.14, 1.45)* | 1.31 (1.15, 1.49)* | 1.21 (1.05, 1.41)* |  | 2.21 (1.75, 2.78)* | 1.96 (1.44, 2.65)* | 1.54 (1.18, 2.01)* |
| Birth order |  |  |  |  |  |  |  |  |  |  |  |  |
| Only child | 49097 | 1.00 (Ref) | 1.00 (Ref) | 1.00 (Ref) |  | 1.00 (Ref) | 1.00 (Ref) | 1.00 (Ref) |  | 1.00 (Ref) | 1.00 (Ref) | 1.00 (Ref) |
| Oldest child | 7941 | 1.07 (0.98, 1.17) | 1.02 (0.93, 1.12) | 1.01 (0.91, 1.11) |  | 1.16 (0.99, 1.37) | 1.18 (0.98, 1.41) | 1.18 (0.97, 1.44) |  | 1.71 (1.38, 2.13)* | 1.49 (1.14, 1.95)* | 1.3 (1.02, 1.65)* |
| Middle child | 6696 | 1.02 (0.87, 1.19) | 0.97 (0.81, 1.16) | 1.01 (0.83, 1.23) |  | 1.32 (1.15, 1.51)* | 1.36 (1.18, 1.56)* | 1.24 (1.06, 1.44)* |  | 2.18 (1.70, 2.78)* | 2.02 (1.49, 2.73)* | 1.54 (1.14, 2.08)* |
| Youngest child | 3162 | 1.22 (1.06, 1.41)* | 1.25 (1.04, 1.50)* | 1.28 (1.06, 1.54)* |  | 1.61 (1.29, 2.01)* | 1.62 (1.26, 2.09)* | 1.53 (1.20, 1.94)* |  | 1.93 (1.49, 2.52)* | 2.02 (1.57, 2.59)* | 1.67 (1.28, 2.17)* |
| Number of younger siblings |  |  |  |  |  |  |  |  |  |  |  |  |
| None (only child) | 49097 | 1.00 (Ref) | 1.00 (Ref) | 1.00 (Ref) |  | 1.00 (Ref) | 1.00 (Ref) | 1.00 (Ref) |  | 1.00 (Ref) | 1.00 (Ref) | 1.00 (Ref) |
| One (oldest child) | 3691 | 1.12 (0.98, 1.28) | 1.08 (0.94, 1.24) | 1.09 (0.93, 1.26) |  | 1.03 (0.82, 1.28) | 1.04 (0.78, 1.38) | 1.09 (0.80, 1.49) |  | 1.20 (0.94, 1.53) | 1.12 (0.85, 1.48) | 1.08 (0.78, 1.50) |
| Two or more (oldest child) | 4250 | 1.03 (0.93, 1.13) | 0.96 (0.85, 1.09) | 0.93 (0.81, 1.08) |  | 1.28 (1.05, 1.56)* | 1.29 (1.06, 1.57)* | 1.25 (1.01, 1.54)* |  | 2.17 (1.61, 2.92)* | 1.82 (1.21, 2.73)* | 1.46 (1.02, 2.09)* |
| Number of older siblings |  |  |  |  |  |  |  |  |  |  |  |  |
| None (only child) | 49097 | 1.00 (Ref) | 1.00 (Ref) | 1.00 (Ref) |  | 1.00 (Ref) | 1.00 (Ref) | 1.00 (Ref) |  | 1.00 (Ref) | 1.00 (Ref) | 1.00 (Ref) |
| One (youngest child) | 2968 | 1.23 (1.05, 1.45)* | 1.27 (1.03, 1.55)* | 1.27 (1.02, 1.59)* |  | 1.62 (1.30, 2.01)* | 1.64 (1.30, 2.07)* | 1.59 (1.27, 2.00)* |  | 1.90 (1.45, 2.50)* | 2.01 (1.56, 2.58)* | 1.65 (1.25, 2.19)* |
| Two or more (youngest child) | 194 | 0.96 (0.52, 1.79) | 1.10 (0.59, 2.04) | 1.24 (0.65, 2.36) |  | 1.53 (0.71, 3.31) | 1.97 (0.77, 5.02) | 1.86 (0.69, 4.99) |  | 2.50 (1.17, 5.33)* | 2.72 (0.96, 7.74) | 2.21 (0.80, 6.10) |

Ref: reference category

^a^ Model I: adjusted for age and sex

^b^ Model II: adjusted for age, sex and childhood characteristics (birthweight, feeding patterns, parental age at child birth, workday TV time, internet use time, snacking frequency)

^c^ Model III: adjusted for age, sex, childhood characteristics and family characteristics (parental weight status, parental educational level, family income and residential site)

**p* < 0.05

**Table A2** Interaction effect between sibship size or structure and sex for thinness by using multinomial logistical regression

|  | Grade 1 thinness | | |  | Grade 2 thinness | | |  | Grade 3 thinness | | |
| --- | --- | --- | --- | --- | --- | --- | --- | --- | --- | --- | --- |
|  | Model I | Model II | Model III |  | Model I | Model II | Model III |  | Model I | Model II | Model III |
| Number of siblings |  |  |  |  |  |  |  |  |  |  |  |
| None (only child) | 1.00 (Ref) | 1.00 (Ref) | 1.00 (Ref) |  | 1.00 (Ref) | 1.00 (Ref) | 1.00 (Ref) |  | 1.00 (Ref) | 1.00 (Ref) | 1.00 (Ref) |
| One | 1.06 (0.83, 1.34) | 1.05 (0.81, 1.36) | 1.04 (0.78, 1.38) |  | 1.29 (0.82, 2.04) | 1.45 (0.84, 2.50) | 1.39 (0.84, 2.30) |  | 1.40 (0.63, 3.15) | 1.19 (0.46, 3.07) | 1.15 (0.42, 3.16) |
| Two or more | 0.92 (0.71, 1.19) | 0.94 (0.64, 1.38) | 0.98 (0.61, 1.59) |  | 1.62 (1.08, 2.43)* | 1.69 (1.07, 2.69)* | 1.70 (1.04, 2.79)* |  | 2.00 (1.15, 3.48)* | 1.70 (0.76, 3.78) | 1.48 (0.67, 3.23) |
| Number of siblings * sex |  |  |  |  |  |  |  |  |  |  |  |
| None (only child) * sex | 1.00 (Ref) | 1.00 (Ref) | 1.00 (Ref) |  | 1.00 (Ref) | 1.00 (Ref) | 1.00 (Ref) |  | 1.00 (Ref) | 1.00 (Ref) | 1.00 (Ref) |
| One * sex | 1.07 (0.91, 1.26) | 1.07 (0.89, 1.29) | 1.08 (0.89, 1.31) |  | 0.99 (0.75, 1.31) | 0.92 (0.64, 1.32) | 0.95 (0.68, 1.32) |  | 1.05 (0.62, 1.78) | 1.16 (0.63, 2.12) | 1.11 (0.58, 2.15) |
| Two or more * sex | 1.07 (0.90, 1.26) | 1.01 (0.78, 1.32) | 0.99 (0.72, 1.37) |  | 0.86 (0.66, 1.12) | 0.84 (0.64, 1.11) | 0.80 (0.60, 1.07) |  | 1.07 (0.77, 1.48) | 1.09 (0.64, 1.87) | 1.03 (0.61, 1.72) |
| Birth order |  |  |  |  |  |  |  |  |  |  |  |
| Only child | 1.00 (Ref) | 1.00 (Ref) | 1.00 (Ref) |  | 1.00 (Ref) | 1.00 (Ref) | 1.00 (Ref) |  | 1.00 (Ref) | 1.00 (Ref) | 1.00 (Ref) |
| Oldest child | 0.76 (0.54, 1.06) | 0.76 (0.49, 1.16) | 0.73 (0.45, 1.18) |  | 1.59 (1.02, 2.47)* | 1.48 (0.86, 2.55) | 1.70 (0.94, 3.06) |  | 1.31 (0.62, 2.75) | 1.06 (0.48, 2.32) | 1.11 (0.51, 2.41) |
| Middle child | 1.24 (0.85, 1.81) | 1.29 (0.77, 2.15) | 1.40 (0.80, 2.43) |  | 1.41 (0.85, 2.33) | 1.51 (0.78, 2.90) | 1.47 (0.79, 2.71) |  | 2.03 (1.20, 3.44)* | 1.87 (0.78, 4.49) | 1.48 (0.59, 3.71) |
| Youngest child | 0.97 (0.71, 1.34) | 1.00 (0.70, 1.42) | 1.01 (0.70, 1.48) |  | 1.42 (0.74, 2.70) | 1.84 (0.83, 4.06) | 1.52 (0.68, 3.35) |  | 1.61 (0.78, 3.34) | 1.31 (0.54, 3.17) | 1.17 (0.44, 3.06) |
| Birth order * sex |  |  |  |  |  |  |  |  |  |  |  |
| Only child * sex | 1.00 (Ref) | 1.00 (Ref) | 1.00 (Ref) |  | 1.00 (Ref) | 1.00 (Ref) | 1.00 (Ref) |  | 1.00 (Ref) | 1.00 (Ref) | 1.00 (Ref) |
| Oldest child * sex | 1.23 (1.03, 1.48)* | 1.20 (0.93, 1.55) | 1.22 (0.90, 1.65) |  | 0.82 (0.63, 1.08) | 0.87 (0.60, 1.25) | 0.80 (0.54, 1.19) |  | 1.18 (0.70, 2.00) | 1.24 (0.71, 2.16) | 1.10 (0.66, 1.85) |
| Middle child * sex | 0.87 (0.66, 1.15) | 0.82 (0.58, 1.17) | 0.80 (0.55, 1.15) |  | 0.96 (0.70, 1.31) | 0.93 (0.62, 1.40) | 0.89 (0.61, 1.29) |  | 1.05 (0.79, 1.38) | 1.05 (0.62, 1.79) | 1.03 (0.58, 1.82) |
| Youngest child * sex | 1.17 (0.94, 1.44) | 1.17 (0.93, 1.47) | 1.17 (0.91, 1.52) |  | 1.09 (0.72, 1.66) | 0.92 (0.51, 1.68) | 1.01 (0.55, 1.84) |  | 1.13 (0.72, 1.77) | 1.34 (0.75, 2.39) | 1.28 (0.68, 2.42) |
| Number of younger siblings |  |  |  |  |  |  |  |  |  |  |  |
| None (only child) | 1.00 (Ref) | 1.00 (Ref) | 1.00 (Ref) |  | 1.00 (Ref) | 1.00 (Ref) | 1.00 (Ref) |  | 1.00 (Ref) | 1.00 (Ref) | 1.00 (Ref) |
| One (oldest child) | 1.03 (0.64, 1.67) | 0.98 (0.53, 1.82) | 0.95 (0.51, 1.78) |  | 0.82 (0.42, 1.59) | 0.76 (0.39, 1.49) | 0.96 (0.49, 1.88) |  | 1.02 (0.33, 3.20) | 0.91 (0.29, 2.84) | 0.93 (0.28, 3.12) |
| Two or more (oldest child) | 0.54 (0.29, 0.98) | 0.54 (0.31, 0.96) | 0.50 (0.25, 1.01) |  | 2.58 (1.46, 4.55)* | 2.50 (1.21, 5.18)* | 2.71 (1.20, 6.13)* |  | 1.66 (0.81, 3.37) | 1.26 (0.56, 2.83) | 1.32 (0.58, 3.01) |
| Number of younger siblings * sex |  |  |  |  |  |  |  |  |  |  |  |
| None (only child) * sex | 1.00 (Ref) | 1.00 (Ref) | 1.00 (Ref) |  | 1.00 (Ref) | 1.00 (Ref) | 1.00 (Ref) |  | 1.00 (Ref) | 1.00 (Ref) | 1.00 (Ref) |
| One (oldest child) * sex | 1.05 (0.81, 1.38) | 1.06 (0.75, 1.52) | 1.09 (0.76, 1.55) |  | 1.15 (0.76, 1.72) | 1.21 (0.80, 1.83) | 1.08 (0.70, 1.67) |  | 1.10 (0.54, 2.26) | 1.14 (0.56, 2.30) | 1.09 (0.50, 2.40) |
| Two or more (oldest child) * sex | 1.47 (1.06, 2.06)* | 1.41 (1.01, 1.96)* | 1.44 (0.95, 2.19) |  | 0.65 (0.45, 0.92) | 0.66 (0.41, 1.06) | 0.62 (0.37, 1.02) |  | 1.18 (0.72, 1.94) | 1.25 (0.72, 2.18) | 1.06 (0.64, 1.77) |
| Number of older siblings |  |  |  |  |  |  |  |  |  |  |  |
| None (only child) | 1.00 (Ref) | 1.00 (Ref) | 1.00 (Ref) |  | 1.00 (Ref) | 1.00 (Ref) | 1.00 (Ref) |  | 1.00 (Ref) | 1.00 (Ref) | 1.00 (Ref) |
| One (youngest child) | 1.06 (0.73, 1.53) | 1.08 (0.70, 1.65) | 1.07 (0.68, 1.68) |  | 1.58 (0.81, 3.06) | 2.10 (0.89, 4.91) | 1.73 (0.78, 3.85) |  | 1.54 (0.69, 3.43) | 1.20 (0.42, 3.47) | 1.09 (0.36, 3.37) |
| Two or more (youngest child) | 0.21 (0.01, 5.00) | 0.23 (0.01, 8.38) | 0.28 (0.01, 10.92) |  | 0.22 (0.02, 2.45) | 0.19 (0.01, 4.09) | 0.29 (0.02, 4.80) |  | 2.50 (0.15, 41.04) | 3.76 (0.14, 102.34) | 2.82 (0.12, 65.75) |
| Number of older siblings * sex |  |  |  |  |  |  |  |  |  |  |  |
| None (only child) * sex | 1.00 (Ref) | 1.00 (Ref) | 1.00 (Ref) |  | 1.00 (Ref) | 1.00 (Ref) | 1.00 (Ref) |  | 1.00 (Ref) | 1.00 (Ref) | 1.00 (Ref) |
| One (youngest child) * sex | 1.11 (0.89, 1.39) | 1.12 (0.87, 1.44) | 1.13 (0.86, 1.48) |  | 1.02 (0.67, 1.56) | 0.85 (0.45, 1.58) | 0.95 (0.52, 1.71) |  | 1.15 (0.69, 1.92) | 1.41 (0.71, 2.81) | 1.33 (0.64, 2.73) |
| Two or more (youngest child) * sex | 2.87 (0.50, 16.58) | 2.83 (0.37, 21.76) | 2.67 (0.34, 20.99) |  | 3.72 (0.76, 18.22) | 4.42 (0.78, 25.13) | 3.35 (0.73, 15.45) |  | 1.01 (0.16, 6.3) | 0.78 (0.09, 6.88) | 0.83 (0.10, 6.64) |

Ref: reference category

a Model I: adjusted for age, sex

b Model II: adjusted for age, sex and childhood characteristics (birthweight, feeding patterns, parental age at child birth, workday TV time, internet use time, snacking frequency)

c Model III: adjusted for age, sex, childhood characteristics and family characteristics (parental weight status, parental educational level, family income and residential site)

*p < 0.05
